# Functional analysis of a novel ENU-induced PHD finger 11 (*Phf11)* mouse mutant

**DOI:** 10.1007/s00335-014-9535-x

**Published:** 2014-08-05

**Authors:** Youming Zhang, Charlotte Dean, Lauren Chessum, Dao Nguyen, Michelle Stewart, Martin Taylor, William O. Cookson, Miriam F. Moffatt

**Affiliations:** 1 Molecular Genetics and Genomics Group, Division of Respiratory Sciences, National Heart and Lung Institute, Imperial College London, Dovehouse Street, London, SW3 6LY UK; 2 Leukocyte Biology section, National Heart and Lung Institute, Imperial College London, London, UK; 3 MRC Harwell, Harwell, Oxfordshire OX11 0RD UK; 4 Wellcome Trust Centre for Human Genetics, University of Oxford, Oxford, UK

## Abstract

Previously, human genetic studies have shown association between polymorphisms within the gene encoding plant homeodomain zinc finger protein 11 (PHF11) and asthma-related phenotypes. Initial functional studies have suggested that PHF11 may be involved in the immune response through regulation of T cell activities. In order to study further the gene’s functions, we have investigated the mouse *Phf11* locus. We have established and characterised a mouse line harbouring a point mutation in the PHD domain of Phf11. Full-length mouse cDNA for *Phf11* was obtained by applying rapid amplification of cDNA ends (RACE). All five exons encoding the PHD domain of *Phf11* were directly sequenced in 3840 mouse DNA samples from the UK MRC Harwell ENU (*N*-ethyl-*N*-nitrosourea)-mutagenised DNA archive. Mice harbouring a valine to alanine substitution, predicted to have a significant functional impact on the PHD zinc finger domain, were re-derived. These Phf11 mutant mice were outcrossed to C3H mice and then backcrossed for ten generations in order to establish a congenic line harbouring the single point mutation in *Phf11*. Macroscopic examination, haematology and histological examination of lung structure revealed no significant differences between mutant and wild-type mice. After administration of lipopolysaccharide, the level of expression of *Il2*, *NF-kB* and *Setdb2* were significantly increased in *Phf11* mutant homozygous lungs compared to control littermates. Our results provide evidence that Phf11 can operate as a Th1 cell regulator in immune responses. Moreover, our data indicate that these mice may provide a useful model for future studies on Phf11.

**Electronic supplementary material:**

The online version of this article (doi:10.1007/s00335-014-9535-x) contains supplementary material, which is available to authorised users.

## Introduction

Asthma is one of the most common diseases in both developed and developing countries. It is characterised by intermittent inflammation of the large airways of the lung with symptoms of wheeze and shortness of breath. Asthma is a complex disease, caused by a combination of genetic and environmental factors. We have previously, through positional cloning, established that polymorphisms of *PHF11* on human chromosome 13 are associated with asthma susceptibility and the asthma-related trait of total serum immunoglobulin E concentration (IgE) (Zhang et al. 2003). Initial functional studies by others (Clarke 2008) suggested that PHF11 may have a role in the regulation of T-cell activities, although the precise functional role(s) of PHF11 in asthma pathogenesis is as yet undetermined.

Mouse models are powerful experimental tools for the study of complex diseases such as asthma, not only for the mapping of disease-associated quantitative traits (De Sanctis et al. 1995; Zhang et al. 1999), but also to dissect the function of novel genes. One of the methods employed for functional analysis is *N*-ethyl-*N*-nitrosourea (ENU)-induced mutagenesis of the mouse genome. Both gene-driven and phenotype-driven approaches have been successfully carried out using ENU mutagenesis (Yates et al. 2009). The establishment of large archives of DNA and frozen sperm from the F1 progeny of mutagenised mice, such as the UK MRC Harwell archive (http://www.har.mrc.ac.uk/services/enu-dna-archive) has facilitated the identification of mutations in any given gene of interest within the mouse genome. In order to investigate the function of Phf11 in vivo, we first generated a full-length cDNA of *Phf11* and using it established the gene copy numbers of *Phf11* in the mouse genome. Subsequently, RNA expression of the mouse *Phf11* gene was investigated using multiple tissue cDNA panels. Following this, we went on to screen the UK MRC Harwell archive of ENU-mutagenised F1 DNAs for mutations in the PHD domain of *Phf11*.

We sequenced the first five exons of *Phf11* that encode the PHD zinc finger functional domain and found a number of different mutations. Focusing on a single *Phf11* ENU-induced mutation bioinformatically predicted to have the greatest impact on gene function, we generated mice and performed phenotypic characterization.

## Methods

### Full-length cDNA identification and multiple tissue expression

The murine homologous locus of human *PHF11* lies on mouse chromosome 14.We searched Ensemble database and revealed that EMBL BC030186 cDNA sequence had the highest similarity to human *PHF11* cDNA. It was therefore used as an initial reference sequence for *Phf11* cDNA. Using this reference sequence, PCR was performed using multiple mouse cDNA tissues (CLONTECH). PCR products of expected size were sequenced (Big Dye Terminator sequencing, Applied Biosystems) allowing a modification and a more accurate *Phf11* cDNA sequence to be derived. Based on this modified cDNA sequence, full-length *Phf11* cDNA was cloned following 5′ and 3′ RACE with mouse spleen Marathon-Ready cDNA (CLONTECH). This was then used as the positive control for expression screening of the mouse multiple tissues cDNA panel 1 (CLONTECH). Amplification was conducted following manufacture’s protocol using the forward primer: ^5′^AGAGGCCACTGAAAGTGCTGATGACC^3′^, and reverse primer: ^5′^CTGCCTTTGCCTTTCTTCCCATTCAC^3′^. Marathon-Ready cDNA RACE libraries (CLONTECH) were then used to extend 5′ and 3′ cDNA ends. Distinct bands from RACE PCR gels were excised, purified and subsequently cloned using PCR Cloning kit (Invitrogen). The inserts were sequenced using Big Dye Terminator sequencing with an ABI 3700 sequencer (Life Technologies^TM^).

### Screening the UK MRC mutagenesis archive

To identify mutations in *Phf11*, total 3840 mutagenised BALB/C DNA samples from the UK MRC Harwell ENU archive were screened by PCR. Primers were designed to target the region encoding the PHD zinc finger domain (exons 2–5) likely to be critical for function. PCR primer sequences used are listed in Supplemental Table S1.

Thirty-five PCR cycles consisting of 60 s at 94 °C, followed by 60 s at 50–60 °C and 30 s at 72 °C were performed. PCR products were purified and sequenced (forward and reverse) as described above. Mutations were identified by comparing with wild-type BALB/c sequence data. Non-synonymous mutations in the PHD zinc finger domain were analysed using the PFAM Hidden Markov Model (HMM).

### Mouse line recovery and maintenance

Mice were housed and maintained in accordance with the rules and regulations of the UK Home Office and the Harwell ethical review committee. Sperm containing mutation Val103Ala was recovered from the Harwell archive and used for in vitro fertilization (IVF) with C3H females to obtain live mice. To identify mice carrying the *Phf11* mutation, progeny born following IVF were genotyped using a diagnostic restriction enzyme digest followed by PCR. Mouse ear clips tissue were digested overnight at 55 °C in 200 µl of lysis buffer consisting of 50 mM Tris–HCL (pH 8.5), 1 mM EDTA and 0.5 % Tween with the addition of proteinase K at a final concentration of 4 mg/ml. After digestion, the proteinase K was heat-inactivated by incubation at 100 °C for 12 min. One µl of the resulting DNA sample was used in the subsequent PCR reaction. PCR conditions were 35 cycles consisting of 60 s at 94 °C, followed by 60 s at 55 °C and then 30 s at 72 °C. PCR products were digested with *Sac II* (NEB Ltd) (2U for 3 h) and then resolved on 2 % agarose gels. Digest product sizes were a single 397 bp product for wild-type and two separate products of 195 and 202 bp for mutant. A congenic *Phf11* line was then established by backcrossing with CH3 mice for ten generations.

### Haemoanalysis, histology and immunostaining

Total blood cell counts as well as percentages of neutrophils, monocytes, eosinophils and lymphocytes were determined for Phf11 homozygotes and wild-type littermates.

Four-micrometre paraffin sections of lung tissue were stained with haematoxylin and eosin for histological examination. Immunostaining with antibodies against aquaporin-5; 1:400, CC-10; 1:500, Santa Cruz, pro-SP-C; 1:1000, Chemicon and alpha smooth muscle actin; 1:1000 Lab Vision was also performed. Incubation in primary antibodies was carried out overnight at 4 °C using previously established protocols. For CC10 immunostaining, antigen retrieval was required to unmask antigens prior to immunodetection. Antibodies were detected using the ABC elite staining kit (Vector Labs).

### Lipopolysaccharides (LPS) administration and qPCR analysis

*Phf11* homozygous mutant and wild-type mice were challenged once intranasally with 10 µg LPS (in PBS)/mouse. Mice were sacrificed 24 h post challenge and broncho-alveolar lavage fluid (BALF), blood and lung tissue were obtained for further analysis.

Total RNA, from mouse lungs, was extracted using Qiagen RNeasy maxi columns (Qiagen), according to manufacturer’s protocol. cDNA synthesis was carried out using SuperScript^®^ III Reverse Transcriptase (Life Technologies).

Quantitative real-time RT–PCR (qPCR) was conducted using Taqman (Life Technologies^TM^) primers (primer details available on request). All measurements were performed in three replicates for each gene(*Il13, Il5, Il12r, Il12ra, Il2rb, Il2, Tnfa, Ifng* and *Nfkb*) with *Gapdh* as the reference gene (*n* = 4 wild-type and *n* = 4 Phf11 homozygotes). Alterations in gene expression in mutant mice were calculated relative to the mean levels in wild-type, the latter being given a standardized value of one. Negative controls of reactions without cDNA template were included. Data were analyzed using unpaired *t*-test (Prism 5 for Windows from GraphPad Software Inc). Data presented are averages ± SEM (standard error of the mean).

## Results

### Structure of *Phf11* on mouse chromosome 14 and gene expression in tissues

**Fig. 1:**
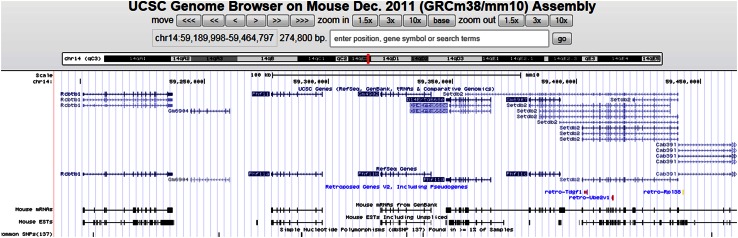
The mouse *Phf11* Locus (from the UCSF Genome Browser). The UCSC Genome Browser view of mouse *Phf11* locus on Dec 2011(GRCm8/mm10) assembly. The genome structure was based on mouse chromosome 14 (chr14:59,189,998-59,464,797274,800 bp). The five paralogous copies of mouse *Phf11* were showed as in Refseq genes*: Phf11c, Phf11d, Phf11b, phf11a* and *Gm6904*

**Fig. 2:**
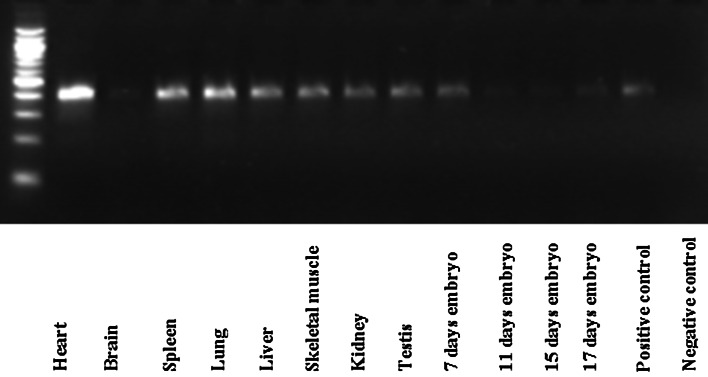
*Phf11* expression in multiple mouse cDNA Tissues. PCRs were performed with multiple tissues mouse cDNAs. The clontech MTC multiple tissue cDNA panels are sets of first-strand cDNAs from 5 to 12 different tissues. The PCR products covered exons 2–5 for mouse *Phf11.* The 100 bp DNA ladder was shown in the first lane

RACE experiments resulted in a 1546 base pair full-length mouse *Phf11* cDNA being obtained as well as a co-transcript consisting of *Setdb2* and *Phf11* (2976 bp). Alignment of the *Phf11* cDNA with the mouse genome revealed multiple hits on chromosome 14. There were five paralogous copies of mouse *Phf11* flanked together over a 148 kb genomic distance. By comparison with the UCSC genome browser (mm10 assembly) (Fig. 1), copies 1, 2, 3, 4 and 5 corresponded to the reference genes *Phf11c*, *Phf11d*, *Phf11b*, *Phf11a* and *Gm6904, respectively*. Further sequence investigation revealed that only the second copy of *Phf11* (corresponding to *Phf11d*) was present in multiple mouse tissue cDNAs (Fig. 2). The exonic structure of *Phf11* copies *Phf11c*, *Phf11b*, *Phf11a*, *Gm6904* showed 80–90 percent similarity compared to *Phf11d*. The remaining divergent sequence in copies *Phf11c*, *Phf11b*, *Phf11a* and *Gm6904* consisted of either duplicated or omitted exons. This suggests that these copies might be pseudogene structures retained in the genome. *Phf11d* contains ten exons with the start codon in exon two and the full-length transcript encoding 337 amino acids. The co-transcript of *Setdb2* and *Phf11* together (2976 bp) was found to be a 22-exon product consisting of *Phf11*c minus the first exon plus all exons of *Setdb2*. *Setdb2* is 9 kb downstream of *Phf11*.

Mouse multiple tissue cDNA panel 1 (CLONTECH) revealed that *Phf11* was present in all adult mouse tissues although expression was noticeably weaker in brain (Fig. 2). We compared the expression levels of *Phf11* throughout mouse embryogenesis and found that *Phf11* was present at embryonic day 7 and then expressed at low levels up to embryonic day 15, after which expression then gradually increased as embryogenesis proceeded.

### Identifying and validating mutagenesis mutations in *Phf11*

BLAST analysis (http://blast.ncbi.nlm.nih.gov/Blast.cgi) with the conserved domain data of National Center for Biotechnology Information (NCBI), revealed that PHD zinc finger protein domain of mouse Phf11 consisted of amino acids 21–167 encoded by exons 2–5 of the *Phf11* cDNA. PCR primers were therefore designed for these exons, and 3840 mutagenised BALB/C DNA samples from the UK MRC Harwell ENU archive were screened. From this initial screening, we identified several *Phf11* mutations which we then went on to validate by repeated sequencing of the DNAs with both forward and reverse primers.

**Fig. 3:**
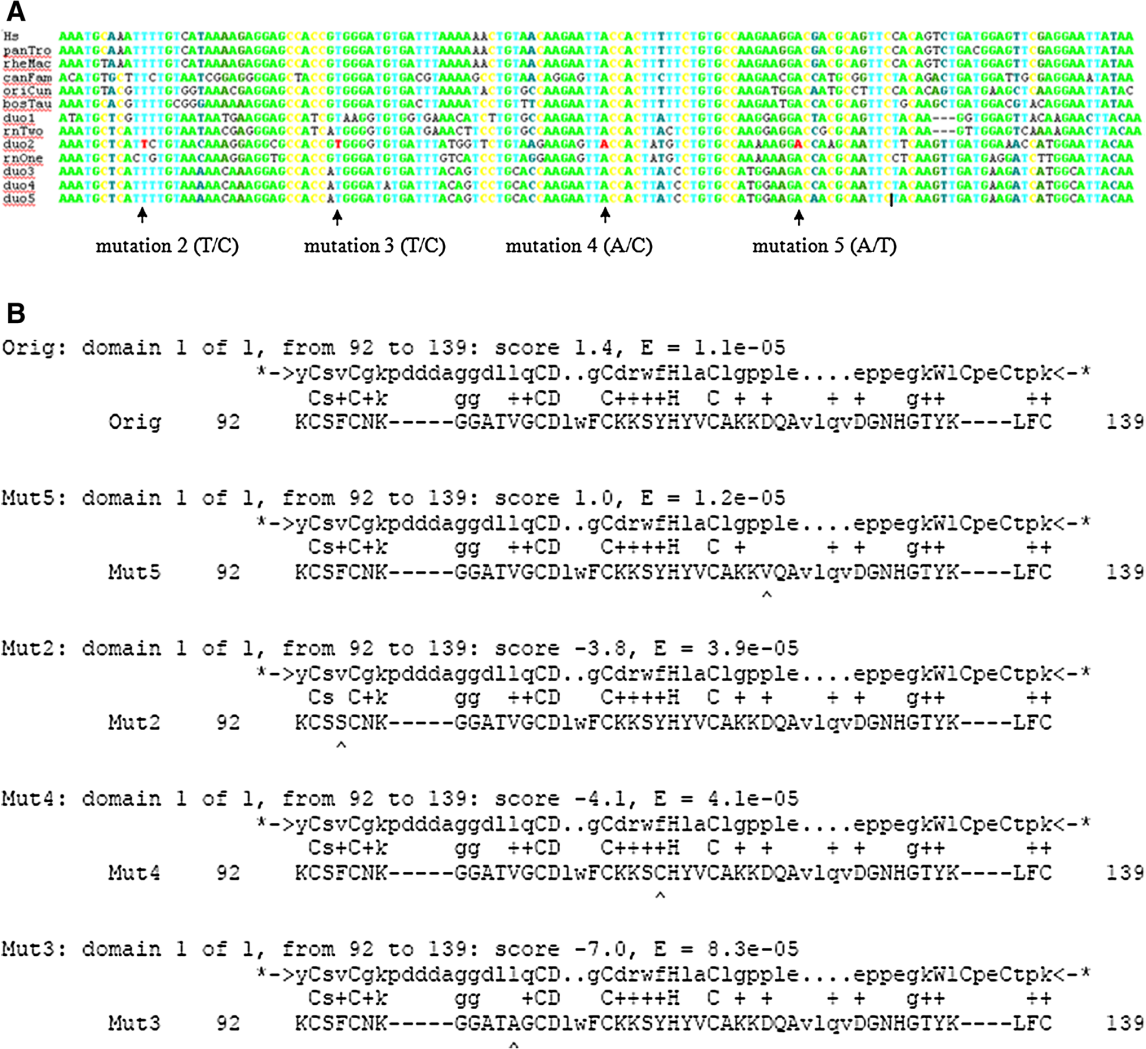
**A** Cross-species conservation analysis for four mutations in the PHD Domain of *Phf11.* Exon 4 multiple alignment (human, chimp, macaque, dog, rabbit, cow, two rat homologs and five mouse duplicates). Mutated residues coloured *red* and change shown in *brackets* with wild-type allele listed first. Each of the mutated residues is conserved in all of the aligned sequences. The mutation-screened paralog (shown as “duo2” here) is not any closer to the ancestral sequence than any of the other paralogos. **B** Predicted impact of identified mutations in PHD domain of *Phf11* on protein function PFAM Hidden Markov Model (HMM) of the PHD domain (hmmbuild trained with PFAM seed alignment and calibrated using hmmcalibrate) was used to score the impact of each substitution. *Mut* mutation. Dn/Ds calculations for this exon were consistent with general selective constraint. Mut5: domain 1 of 1, from 92 to 139: score 1.0, E = 1.2e–05; Mut2: domain 1 of 1, from 92 to 139: score −3.8, E = 3.9e–05; Mut4: domain 1 of 1, from 92 to 139: score −4.1, E = 4.1e–05; Mut3: domain 1 of 1, from 92 to 139: score −7.0, E = 8.3e–05

Five separate mutations in *Phf11* were verified. Mutation 1 was a synonymous mutation in exon 2 (Cys28Cys). The other four mutations were all non-synomous mutations in exon 4: mutation 2- phenylalanine to serine (Phe95Ser); mutation 3- valine to alanine (Val103Ala); mutation 4- tyrosine to cysteine (Tyr114Cys); mutation 5- asparagine to valine (Asp122Val). Mutations 2–5 were also found to be conserved in a cross-species conservation analysis (Fig. 3A).

Using a PFAM Hidden Markov Model of the PHD domain (hmmbuild trained with PFAM seed alignment and calibrated using hmmcalibrate) (Sonnhammer et al. 1998), we scored the impact of each amino acid substitution (mutations Phe95Ser, Val103Ala, Tyr114Cys and Asp122Val). Based on this analysis, each of the non-synonymous mutations identified resulted in an imperfect match to the PHD model (reduction in bit score and e-value). By this measure, all four non-synonymous changes would be expected to impact on protein function. Mutant Val103Ala was predicted to cause the most detrimental change, followed by mutant Tyr114Cys, Phe95Ser and then mutant Asp122Val (Fig. 3B).

### Recovery and maintenance of *Phf11* mutant mice

To establish the mice carrying mutation Val103Ala in *Phf11*, we recovered the parallel sperm sample from the F1 male containing the mutation from the Harwell ENU archive. IVF was subsequently performed using C3H embryos to facilitate genotyping of the mutation that had been induced in the BALB/C mice. Nine offsprings were obtained, six of which contained the mutant allele indicating that a single copy of the mutation did not affect mouse viability or fertility. We subsequently established a congenic *Phf11* line by backcrossing these mice to CH3 for ten generations; thereby ensuring that the line did not contain any additional ENU mutations that might have been present in earlier generations. *Phf11* heterozygotes were inter-crossed and the genotypes of their offspring were analysed at E18.5. Normal Mendelian ratios of mutant versus wild-type offspring were observed, indicating no prenatal mortality and this was confirmed by Chi-squared analysis. Careful observation of *Phf11* homozygotes did not reveal any visible phenotype.

### Haemoanalysis and histological examination of *Phf11* mouse lungs

**Fig. 4:**
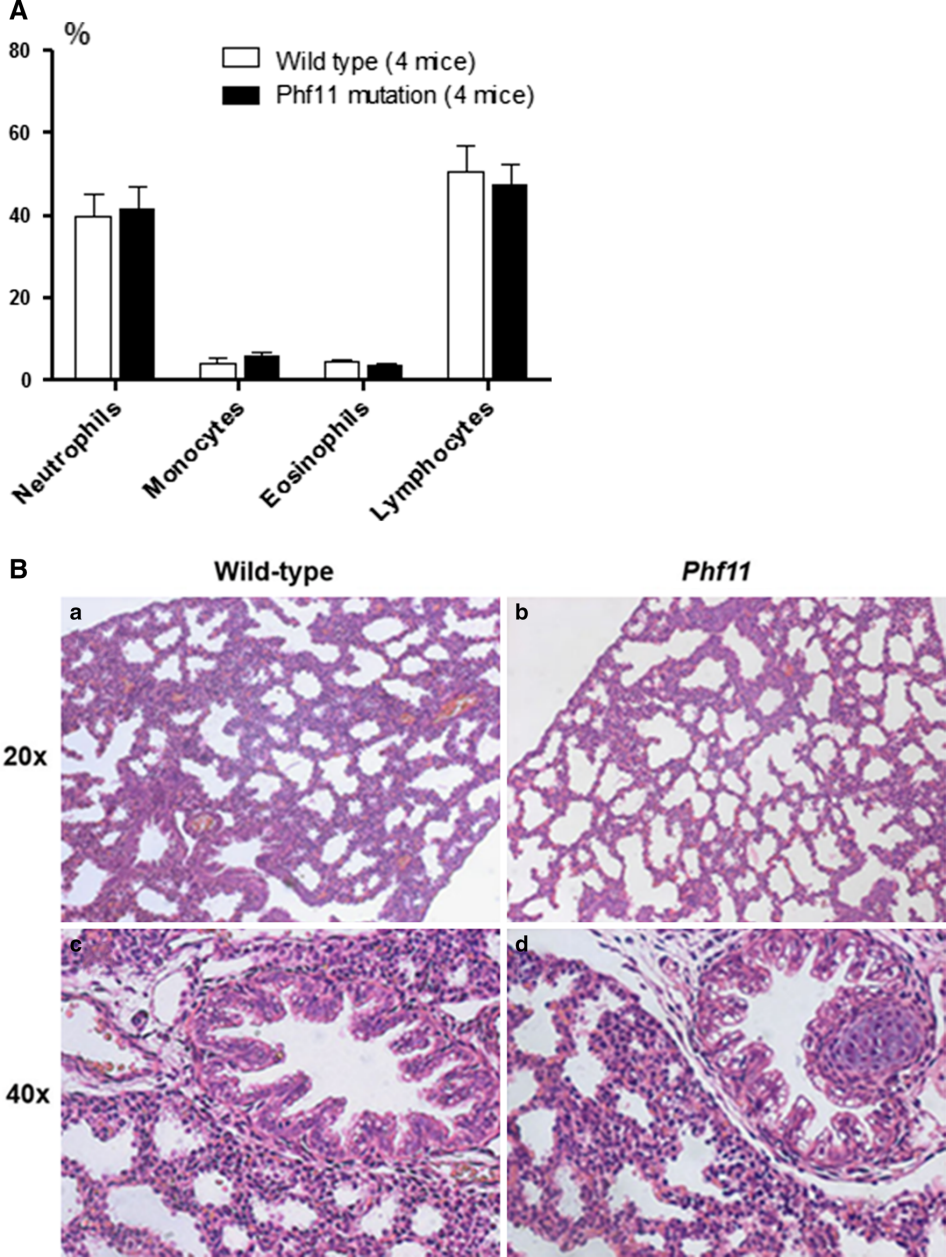

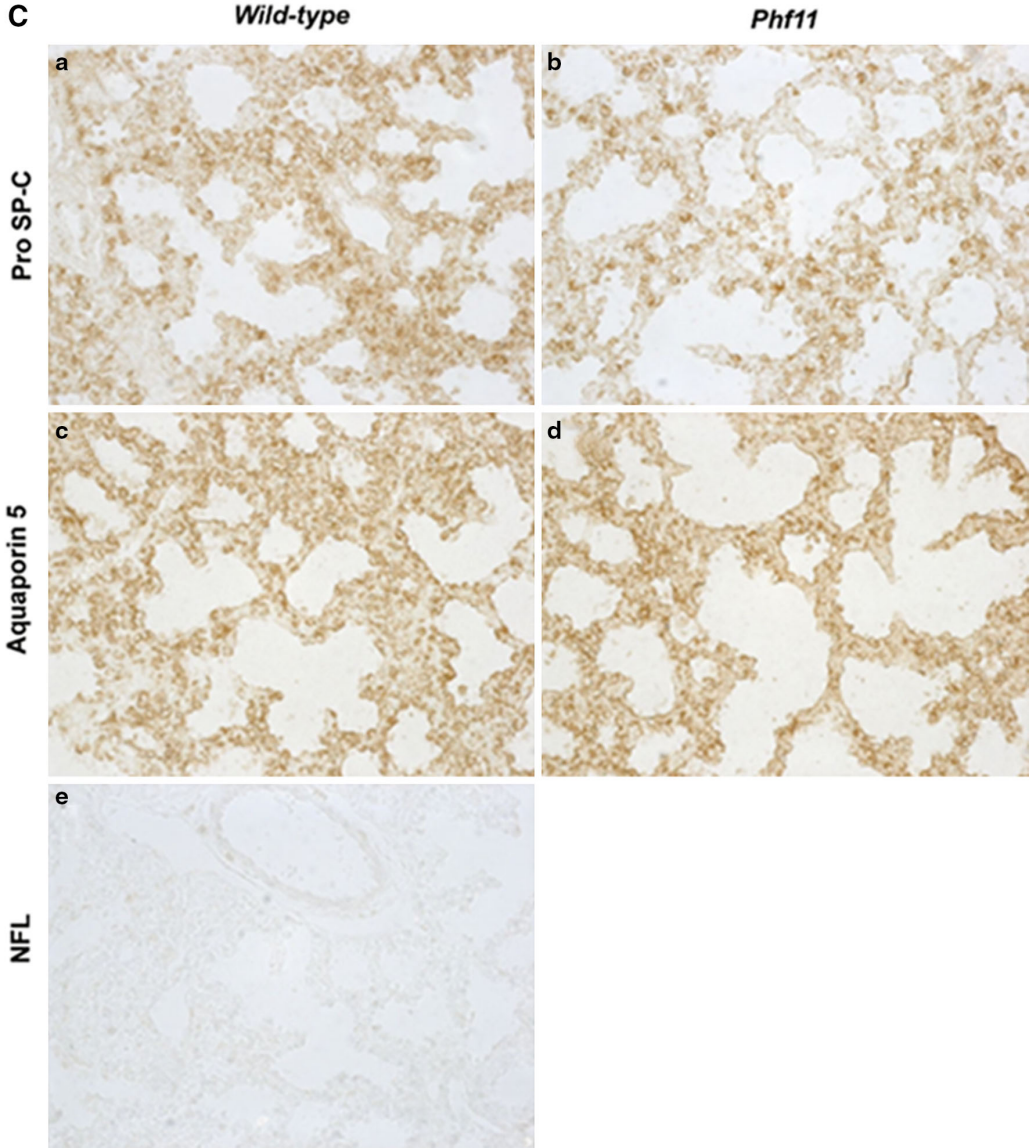
**A** Haemoanalysis in Mouse Blood. **B** Histological comparison of E18.5 Wild-type and *Phf11* homozygous lungs. No difference in either lung parenchyma (*a, b*) or proximal airways (*c, d*) was observed in H&E stained sections of wild-type (*a, c*) and *Phf11* homozygous (*b, d*) lungs. **C** Comparison of Cell-type specific markers in Wild-type and *Phf11* homozygous lungs. No significant difference was observed in expression of the Type II alveolar cell marker pro SP-C (*a, b*) and Type I alveolar cell marker aquaporin 5 (*c, d*) between wild-type (*a, d, e*) and *Phf11* homozygous (*b, d*) lungs. *e* Control lung section with primary antibody omitted

Analysis of total cell numbers in blood from *Phf11* homozygotes and wild-type littermates revealed no significant difference. We then examined specific cell populations. Although the percentage of neutrophils and monocytes was higher and the percentage of lymphocytes lower in *Phf11* homozygotes compared to wild-type, these differences were not statistically significant (Fig. 4A).

To determine whether lung embryogenesis was affected in *Phf11* mutants, we first conducted a macroscopic analysis of homozygous and heterozygous mice compared to wild-type littermate mice. No visible difference was found between genotypes. We then examined the histology of homozygous and wild-type mouse lungs and again found no obvious differences (Fig. 4B, C). Similarly, no significant difference in the expression of a number of cell-type specific markers (pro-Surfactant protein-C, Clara Cell-10 protein, α–Smooth muscle actin and Aquaporin-5) (Fig. 4B and data not shown) was found between *Phf11* homozygotes and wild-type mice indicating that differentiation was not disrupted. These results indicate that the *Phf11* mutation does not affect lung development.

### Analysis of the immune response in *Phf11* homozygous lungs following LPS challenge

Phf11 has previously been suggested to regulate Th1-type cytokine gene expression (Clarke 2008). We therefore investigated whether the Val103Ala mutation in exon 4 of *Phf11* had any influence on the expression of key immune system regulators. Wild-type (*n* = 4) and homozygous mutant (*n* = 4) mice were challenged intranasally with 10 µg LPS (in PBS)/mouse to stimulate the innate immune system. Mice were sacrificed 24 h after dosing, and blood, BALF and lung tissue were obtained.

Transcript levels of *Phf11, Setdb2* and genes encoding either Th1/Th2 cytokines or regulators of airway inflammation were investigated by quantitative PCR. No changes in expression levels were detected in either the BALF or blood samples of *Phf11* mutant mice compared to wild-type mice.

**Fig. 5:**
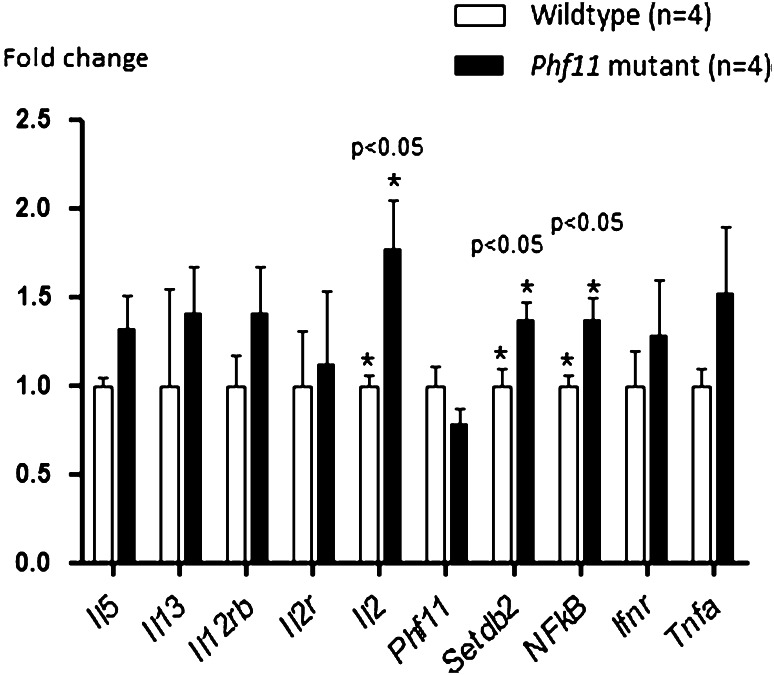
Quantitative PCR analysis expression levels of ten genes in *phf11* mutant mouse lungs after LPS challenge. Alterations in gene expression in *Phf11* homozygous lungs were expressed relative to the mean intensity in wild-type embryos, which was given a standardised value of one. *P* values were compared by Student’s *t*-test. *Error bars* represent SEM and significance was scored using unpaired two-tailed *t*-tests. *indicates statistical significance between wild-type and *Phf11* homozygous mice

Using RNA extracted from mouse lung tissue samples revealed, however, that *Phf11* transcript levels were lower in *Phf11* homozygous mice compared to wild-type, although this was not statistically significant (mean 0.785 ± 0.09, 95 % CI −0.5477 to 0.1167) (Fig. 5). Levels of the Th1 cytokine *Il2* were significantly increased in *Phf11* homozygous mice (mean 1.77 ± 0.27, 95 % CI 0.084–1.462 *P* < 0.05). In addition, the levels of *NFκB* (mean 1.371 ± 0.1230, 95 % CI 0.04021–0.7012 *P* < 0.05) and of *Setdb2* (mean 1.369 ± 0.1030, 95 % CI 0.0183–0.7192 *P* < 0.05) were increased in *Phf11* mutant mice. Transcriptional levels of *Tnfa* and *Ifnr* were also consistently increased in the mutants although neither was statistically significant (Fig. 5). In contrast, we did not detect any change in the Th2 cytokines *Il5* and *Il13* between homozygous mutant and wild-type mice. These results suggest that Phf11 may function specifically as a mediator of Th1-type cytokine expression. In addition, they indicate that the Phf11 mice reported in this manuscript carry a functional mutation in this gene and may serve as a valuable tool for further investigations of Phf11.

## Discussion

Since the completion of the first genome-wide linkage scan of asthma in 1996 (Daniels et al. 1996), several novel genes that underlie asthma and asthma-associated traits have been identified through positional cloning or whole genome association studies (Zhang et al. 2012). One such gene is *PHF11,* which encodes a plant homeodomain finger protein.

Human *PHF11* has 10 exons and a number of alternative first exons, as well as numerous splice variants in both T and B cells (Zhang et al. 2003). The association between SNPs of *PHF11* and asthma or asthma-associated traits has been investigated in many subsequent studies with the majority confirming a positive association in a number of ethnic populations (Hersh et al. 2007; Gao et al. 2010). The G allele of the SNP *rs1046295*, located in the 3′-UTR of *PHF11,* has also been shown to be preferentially transmitted to children with atopic dermatitis (Jang et al. 2005) as well as showing association with decreased *PHF11* RNA in Th1 cells (Clarke 2008). Electrophoretic mobility shift assays have revealed allele-specific binding of *rs1046295* by the transcription factor Oct-1 (Holt et al. 2011).

Mouse ENU mutagenesis is a powerful tool for studies of human diseases. ENU is effective in inducing gain-of-function as well as complete and partial loss-of-function mutations. The generation of allelic series at new and existing gene loci is a particularly appealing prospect in the analysis of gene function (Coghill et al. 2002). One of the major sites for ENU action in the mouse is spermatogonial stem cells, enabling mutations to be transmitted to the next generation (Yates et al. 2009). Mice harbouring the selected mutation can be re-derived and backcrossed through ten generations with control wild-type mice, ensuring that only one mutation of interest is present in the line (Keays et al. 2006). The mutation rate of ENU is such that even screening a relatively small number of DNA samples, around 2500, gives a greater than 90 % chance of detecting one mutation in any gene of interest (Brown and Hardisty 2003). Mouse *Phf11* locus on chromosome 14 has 5 paralogous copies of *Phf11*. The complex structure makes gene targeting (such as gene knockdown) very difficult and time consuming. Alternatively, the ENU-induced mutagenesis approaches on *Phf11* could provide useful resources for dissecting the functions. The point mutations of *Phf11* induced by ENU could have gain- or loss-of- function if these mutations cause the change or disruption of the protein structure. In this study, we identified and confirmed five ENU-induced mutations in the PHD domain of *Phf11*; four of which were non-synonymous. We then generated a congenic line for carrying the mutation that we had determined analytically to have the most significant potential impact on protein function. Homozygous Phf11 mutant mice appeared grossly normal. Following challenge with LPS, *Phf11* gene expression levels were twenty-two per cent lower in Phf11 homozygous mice compared to wild-type controls. Although not statistically significant, the finding suggests that the mutation might be capable of affecting *Phf11* expression. In addition, ENU-induced mutations frequently do not affect the gene at a transcriptional level; instead they result in disruption at the protein level e.g. generation of a truncated protein or unstable protein. The transcriptional levels of Th2 cytokines *Il5* and *Il13* did not show any significant change in *Phf11* mutant mice lungs. Levels of the Th1 cytokine *Il2* were however significantly increased in *Phf11* mutant mice compared to wild-type controls. We also noted consistent, but not significant, an increase in the expressional levels of *Tnfa* and *Ifnr*.

The functions of zinc finger proteins are diverse. They are involved in DNA recognition, RNA packaging, transcriptional activation, regulation of apoptosis, protein folding and assembly, as well as lipid binding (Laity et al. 2001). Mutations within zinc finger domains have been associated with a number of human diseases (Ahmad et al. 1998; Yang et al. 2004; Lindstrom et al. 2007). The Val103Ala mutation in *Phf11*,which we have identified and characterised in this present study, appears to have an impact on Th1 over-expression after LPS stimulation.

The Th1/Th2 balance hypothesis began in the late 1980s with observations in mice of two subtypes of T-helper cells differing in cytokine secretion patterns and other functions (Mosmann et al. 1986). Th1 cells and the pathway they dominate are mostly reliant on IFN-gamma and IL2 whilst Th2 cells are mostly reliant on IL4 and IL5. Asthma is an inflammatory condition where Th2 cytokines dominate. Th2 cytokines are thought to contribute to asthma pathology through their capacity to promote IgE synthesis, the maturation and activation of mast cells and basophils, as well as eosinophil infiltration (Larche et al. 2003).

Interleukin-2 (IL2) was the first of a series of lymphocytotrophic hormones to be recognised and completely characterised. It is pivotal for the generation and regulation of the immune response (Smith 1988). IL2 is secreted by T cells after antigen binding to the T cell receptor (TCR) and T cells also expresse the IL2 receptor, IL2R. The IL2/IL2R interaction stimulates the growth, differentiation and survival of antigen-selected cytotoxic T cells via activation of specific genes (Stern and Smith 1986). Serum IFN-gamma levels have been shown to be significantly higher in asthmatic subjects reflecting the severity of airway inflammation in atopic asthma (ten Hacken et al. 1998).

The results from our current study provide evidence that Th1 cytokine expression is affected by Phf11 during an LPS-stimulated immune response. Specifically, disruption of the zinc finger domain of Phf11 caused upregulation of *Il2* and *Ifnr* expression.

We also observed increased *NF*-*kB* in *Phf11* mutant homozygous mouse lungs. The activation and nuclear translocation of NF-κB have been previously associated with increased transcription of a number of different cytokines including IL1, IL2, TNFα and IL12. These immune mediators are important components of the innate immune response to invading microorganisms and are required for the ability of inflammatory cells to migrate into areas where NFκB is being activated (Caamano and Hunter 2002). How PHF11 interacts with NF-κB remains unknown, but a stimulation-dependent interaction between PHF11and the p65 subunit of NF-κB has been found in nuclear extracts of Jurkat T cells (Clarke 2008).

We have previously identified co- transcripts of *SETDB2* and *PHF11* in our human studies (Zhang et al. 2003). Here, we have also discovered a *Setdb2*-*Phf11* co-transcript in the mouse. We observed increased *Setdb2* levels in *Phf11* homozygous mutant mice compared to wild-type controls indicating a degree of cooperation between Phf11 and Setdb2. In humans, SETDB2 contains a methyl-CpG binding domain. SET domains modulate gene expression epigenetically through histone H3 lysine methylation. Recent studies have revealed an important role for SETDB2 in chromosome segregation (Falandry et al. 2010). The co-ordination of Phf11 and Setdb2 in the immune response would be worthy of further investigation.

Our findings demonstrate that the PHD zinger finger domain of Phf11 is able to regulate Th1 cytokine expression during an immune response. We have established and characterised a novel mouse mutant for *Phf11* which we propose will be a useful tool for furthering our understanding of *Phf11* function allowing insights into the pathogenesis of inflammatory-mediated diseases such as asthma.

## Electronic supplementary material

Below is the link to the electronic supplementary material.

Supplementary material 1 (DOC 54 kb)

## References

[CR1] Ahmad W, Irvine AD, Lam H, Buckley C, Bingham EA, Panteleyev AA, Ahmad M, McGrath JA, Christiano AM (1998). A missense mutation in the zinc-finger domain of the human hairless gene underlies congenital atrichia in a family of Irish travellers. Am J Hum Genet.

[CR2] Brown SD, Hardisty RE (2003). Mutagenesis strategies for identifying novel loci associated with disease phenotypes. Semin Cell Dev Biol.

[CR3] Caamano J, Hunter CA (2002). NF-kappaB family of transcription factors: central regulators of innate and adaptive immune functions. Clin Microbiol Rev.

[CR4] Clarke E, Rahman N, Page N, Rolph MS, Stewart GJ, Jones GJ (2008). Functional characterization of the atopy-associated gene PHF11. J Allergy Clin Immunol.

[CR5] Coghill EL, Hugill A, Parkinson N, Davison C, Glenister P, Clements S, Hunter J, Cox RD, Brown SD (2002). A gene-driven approach to the identification of ENU mutants in the mouse. Nat Genet.

[CR6] Daniels SE, Bhattacharrya S, James A, Leaves NI, Young A, Hill MR, Faux JA, Ryan GF, le Souef PN, Lathrop GM, Musk AW, Cookson WO (1996). A genome-wide search for quantitative trait loci underlying asthma. Nature.

[CR7] De Sanctis GT, Merchant M, Beier DR, Dredge RD, Grobholz JK, Martin TR, Lander ES, Drazen JM (1995). Quantitative locus analysis of airway hyperresponsiveness in A/J and C57BL/6 J mice. Nat Genet.

[CR8] Falandry C, Fourel G, Galy V, Ristriani T, Horard B, Bensimon E, Salles G, Gilson E, Magdinier F (2010). CLLD8/KMT1F is a lysine methyltransferase that is important for chromosome segregation. J Biol Chem.

[CR9] Gao J, Li W, Willis-Owen SA, Jiang L, Ma Y, Tian X, Moffatt M, Cookson W, Lin Y, Zhang Y (2010). Polymorphisms of PHF11 and DPP10 are associated with asthma and related traits in a Chinese population. Respiration.

[CR10] Hersh CP, Raby BA, Soto-Quiros ME, Murphy AJ, Avila L, Lasky-Su J, Sylvia JS, Klanderman BJ, Lange C, Weiss ST, Celedon JC (2007). Comprehensive testing of positionally cloned asthma genes in two populations. Am J Respir Crit Care Med.

[CR11] Holt RJ, Zhang Y, Binia A, Dixon AL, Vandiedonck C, Cookson WO, Knight JC, Moffatt MF (2011). Allele-specific transcription of the asthma-associated PHD finger protein 11 gene (PHF11) modulated by octamer-binding transcription factor 1 (Oct-1). J Allergy Clin Immunol.

[CR12] Jang N, Stewart G, Jones G (2005). Polymorphisms within the PHF11 gene at chromosome 13q14 are associated with childhood atopic dermatitis. Genes Immun.

[CR13] Keays DA, Clark TG, Flint J (2006). Estimating the number of coding mutations in genotypic- and phenotypic-driven *N*-ethyl-*N*-nitrosourea (ENU) screens. Mamm Genome.

[CR14] Laity JH, Lee BM, Wright PE (2001). Zinc finger proteins: new insights into structural and functional diversity. Curr Opin Struct Biol.

[CR15] Larche M, Robinson DS, Kay AB (2003). The role of T lymphocytes in the pathogenesis of asthma. J Allergy Clin Immunol.

[CR16] Lindstrom MS, Jin A, Deisenroth C, White Wolf G, Zhang Y (2007). Cancer-associated mutations in the MDM2 zinc finger domain disrupt ribosomal protein interaction and attenuate MDM2-induced p53 degradation. Mol Cell Biol.

[CR17] Mosmann TR, Cherwinski H, Bond MW, Giedlin MA, Coffman RL (1986). Two types of murine helper T cell clone. I. Definition according to profiles of lymphokine activities and secreted proteins. J Immunol.

[CR18] Smith KA (1988). Interleukin-2: inception, impact, and implications. Science.

[CR19] Sonnhammer EL, Eddy SR, Birney E, Bateman A, Durbin R (1998). Pfam: multiple sequence alignments and HMM-profiles of protein domains. Nucleic Acids Res.

[CR20] Stern JB, Smith KA (1986). Interleukin-2 induction of T-cell G1 progression and c-myb expression. Science.

[CR21] ten Hacken NH, Oosterhoff Y, Kauffman HF, Guevarra L, Satoh T, Tollerud DJ, Postma DS (1998). Elevated serum interferon-gamma in atopic asthma correlates with increased airways responsiveness and circadian peak expiratory flow variation. Eur Respir J.

[CR22] Yang F, Yamashita J, Tang E, Wang HL, Guan K, Wang CY (2004). The zinc finger mutation C417R of I-kappa B kinase gamma impairs lipopolysaccharide- and TNF-mediated NF-kappa B activation through inhibiting phosphorylation of the I-kappa B kinase beta activation loop. J Immunol.

[CR23] Yates L, McMurray F, Zhang Y, Greenfield A, Moffatt M, Cookson W, Dean C (2009). ENU mutagenesis as a tool for understanding lung development and disease. Biochem Soc Trans.

[CR24] Zhang Y, Lefort J, Kearsey V, Lapa e Silva JR, Cookson WO, Vargaftig BB (1999). “A genome-wide screen for asthma-associated quantitative trait loci in a mouse model of allergic asthma.”. Hum Mol Genet.

[CR25] Zhang Y, Leaves NI, Anderson GG, Ponting CP, Broxholme J, Holt R, Edser P, Bhattacharyya S, Dunham A, Adcock IM, Pulleyn L, Barnes PJ, Harper JI, Abecasis G, Cardon L, White M, Burton J, Matthews L, Mott R, Ross M, Cox R, Moffatt MF, Cookson WO (2003). Positional cloning of a quantitative trait locus on chromosome 13q14 that influences immunoglobulin E levels and asthma. Nat Genet.

[CR26] Zhang Y, Moffatt MF, Cookson WO (2012). Genetic and genomic approaches to asthma: new insights for the origins. Curr Opin Pulm Med.

