# Supplementary material for: Functional analysis of a novel ENU-induced PHD finger 11 (Phf11) mouse mutant
Source: Mamm Genome. 2014 Aug 5;25(11):573–82. doi: 10.1007/s00335-014-9535-x (PMC4239810; doi:10.1007/s00335-014-9535-x)
Supplement: Supplementary file 1 — Supplementary material 1 (DOC 54 kb) [file 335_2014_9535_MOESM1_ESM.doc]

**Supplemental Table 1. PCR Primers used for *Phf11* Exon**

|  | **Exon Length (bp)** | **Forward Primer Sequence 5’ to 3’** | **Reverse Primer Sequence 5’ to 3’** | **PCR Product Size (bp)** |
| --- | --- | --- | --- | --- |
| **Exon 1** | 124 | GCAACATCCTGCAGTCTC | TTCAGAGTTGAAAATCTGCC | 286 |
| **Exon 2** | 122 | AGTACTGGGAACAGAACTCA | GGTCCAGGAGTTAAGACTC | 352 |
| **Exon 3** | 108 | GCCTGCATCTTTGTGGC | AGGAGCCCAAGGCTCGA | 393 |
| **Exon 4** | 134 | AAACTCATTTGCATGAACTG | GCTGCCAAATCTAAGTGA | 397 |
| **Exon 5** | 49 | TCAGTGCTTATGAACAAGC | CACTGGGAATGCAGAGCA | 331 |
